# Mitigation of pesticide residue levels in the exposed dermal regions of occupationally exposed farmworkers by use of personal protective equipment

**DOI:** 10.3389/fpubh.2023.1232149

**Published:** 2023-08-31

**Authors:** Summaiya Lari, Janardhan Vanka, Babban Jee, Arun Pandiyan, Praveen Yamagani, Senthil Balakrishnan Kumar, Mohan Naidu, Padmaja Jonnalagadda

**Affiliations:** ^1^ICMR-National Institute of Nutrition, Tarnaka, Osmania University, Hyderabad, India; ^2^Department of Biochemistry, Acharya Nagarjuna University, Guntur, India; ^3^Department of Health Research, Ministry of Health and Family Welfare, Government of India, New Delhi, India

**Keywords:** occupational dermal exposure, hand-wash, patch dosimeter, wipe samples, health risk assessment, safety analysis

## Abstract

Unsafe pesticide handling practices with the limited use of personal protective equipment (PPE) by the Indian farming groups lead to an increased risk of exposure to pesticides. Therefore, a community-based follow-up study based on dosimeters, wipes, and hand-wash technique was carried out to evaluate the dermal exposure to pesticides and to analyze the impact of the usage of PPE on minimizing the exposure among the farmworkers of Rangareddy district, Telangana, India. Risk in terms of hazard quotient (HQ), hazard index (HI), and safety analysis as margins of safety was assessed. Farmworkers averaged 18 years of farming experience and showed resistance to adopting good agricultural practices. Ten pesticide residues were detected in concentrations ranging from 0.000 to 246 mg ml^−1^ in hand-wash, 0.000 to 198.33 ng cm^−2^ in patch dosimeter, and 0.000 to 1,740 ng cm^−2^ in wipe samples collected from farmworkers not using PPE. The second phase includes the intervention study results that revealed a significant reduction both in the concentrations and the number of pesticide residues detected in the hand-wash, patch, and wipe samples of the farmworkers who have used the PPE provided to them (*p* < 0.01). Furthermore, the probabilistic health risk assessment in terms of the HQ values ranged from 0.02 to 1029.82, and HI was >1, suggesting the non-carcinogenic risks associated with dermal exposure to pesticides among them. Additionally, the safety risk assessment in terms of the margin of safety suggests that they follow risky handling practices. The study confirms that farmworkers are exposed to pesticides and emphasizes the significance of using PPE in reducing the risk.

## 1. Introduction

Pesticides are used to enhance crop yields by protecting the crops from pest infestation and to meet the increasing consumer demands. However, improper handling and the careless use of pesticides result in a number of issues, including the release of pesticides into the environment and the possibility of unintended negative effects on human health (1). As a result, determining the exposure and associated risk is significant, especially for groups of farmworkers who are occupationally exposed in the agricultural sector (2). Their exposure occurs at various activities of their involvement in farming activities through various ways such as ingestion, inhalation, ocular, or dermal. Of the several routes, the dermal route of exposure plays a key and significant role in the absorption of pesticide residues into the human body (3). Therefore, the use of personal protective equipment (PPE) gained a lot of importance in providing protection from exposure (4). However, from the Indian context point of view, the use of PPE by the farming community is minimal due to the prevailing tropical climatic conditions, such as heat stress, and if available, sometimes due to its inaccessibility/unaffordability (5).

Furthermore, it is challenging to assess the exact dermal exposure due to various variables that might influence dermal absorption and sampling technique effectiveness (6). Therefore, the risk assessment through the dermal absorption of pesticides is considered an important area of research by many researchers for necessitating the need for the development of different direct/indirect methods and models (7, 8). Of them, the use of patch methods/whole-body dosimeters is among the direct methods for accessing the residues that might penetrate through the exposed dermal regions, while a few other indirect methods include washing, wiping, or skin-stripping techniques (9, 10). However, the European Union and North American countries do follow stringent exposure data for the assessment of risk and also do not authorize/allow the use of any molecule until and unless the submission of appropriate data using an adequate model is made available that can maintain the exposure levels of the operators to the expected level of exposure when the pesticides are used by following good agricultural practices (GAPs) (11). However, in India, the data available on exposure assessment among the Indian farming groups is meager. Moreover, the reported incidents of pesticide poisoning among the Indian farming community occur mostly due to their lack of awareness and knowledge on GAPs, probable toxic effects, and adverse health effects induced due to exposure (12, 13).

The research was carried out earlier on the residues reported in different matrices (14, 15); however, very little information is available on the dermal exposure of pesticide residues before/after the use of PPE among the Indian farming community in the real-time field scenario. Hence, the present investigation was aimed to assess the dermal exposure to pesticides among the selected farmworkers of the Rangareddy district, Telangana, India (a tropical country where the use of plant protection products (PPP) is not only high but where the use of PPE is also limited) so as to obtain systematic data on how the use of PPE mitigates in minimizing the pesticide exposure and potential health risks for farmworkers.

## 2. Materials and methods

### 2.1. Study design and selection of farmworkers

The present community-based follow-up study was conducted by recruiting a total of 120 farmworkers (40 subjects each engaged in paddy, vegetable, and commercial crop cultivation such as cotton) in four identified villages of Rangareddy district, Telangana, India (16). The farmmen and farmwomen between the ages of 18 and 50 years were included, while individuals with dermatological allergies, liver disease/damage, cardiovascular disease, diabetes, hyper/hypotension, and pregnant women were excluded.

The study was conducted in two phases: the first phase included the collection of samples from the farmworkers (*n* = 120) engaged in routine and regular farming activities but not using PPE. The second phase included intervention studies conducted among the same exposed farmworkers to study the impact of the use of PPE provided to them cost-free (coveralls, gloves, boots, goggles, and masks) on exposure. In the second phase of the study, the same subjects were randomly segregated into two groups (*n* = 60) to be provided with PPE cost-free. One group of farmworkers (*n* = 60) was provided with the commercially available PPE as per the EU and EPA norms (4, 17), which includes a Tychem C category III coverall (DuPont™), while farmworkers (*n* = 60) of another group were distributed with PPE prepared using available resources such as empty urea bags (non-absorbent polypropylene material of 50 μm thickness) and cotton lining (absorbent materials) and coverall designed and prepared in-house so as to assess the difference in the use of both the PPE provided and compare the same with those not using PPE, followed by the collection of samples after 90 days of their usage. Both groups were provided with a safety splash goggle, a cup-type respirator, a pair of nitrile gloves, and a pair of polyvinyl chloride (PVC) gumboots cost-free (Usha Fire, DuPont supplier, Hyderabad, India).

### 2.2. Ethical clearance and consent

The study protocol was reviewed and approved by the ethical committee of the Indian Council of Medical Research – National Institute of Nutrition, Hyderabad, India (REF NIN Protocol number 11/I/2016). A written informed consent was obtained from each farmworker, and it was explained to the farmworkers that they could withdraw their participation at any given point in the study period without any prior information/intimation/fine/penalty.

### 2.3. Questionnaire data and field observations

The questionnaire consisted of 172 variables and was pre-tested before its administration to the farmworkers (*n* = 120) in order to obtain the appropriate information (Supplementary file 1). In addition, data on meteorological parameters such as temperature (°C), relative humidity (%), wind velocity (km/h), and direction during their involvement in farming activities were also recorded using a digital anemometer (LM 8010, Lutron Electronic, Taiwan) each time on the day of sample collection, followed by the spraying of pesticides at every point of application for the specific crop in the treated fields.

### 2.4. Chemicals and reagents

The certified reference materials of acephate, chlorpyrifos, dimethoate, emamectin benzoate, imidacloprid, monocrotophos, phenthoate, phorate, profenofos, quinalphos, and triphenyl phosphate (TPP, internal standard) were procured from Sigma-Aldrich Chem. Pvt. Ltd., India, based on the information collected on the types of pesticides used by the farmworkers. The analytical-grade formic acid was procured from Fluka Pvt. Ltd., India, and the other organic solvents, acetonitrile and methanol (pestanal-grade), were obtained from Sigma Aldrich, Merck KGaA Darmstadt, Germany. Salts such as sodium chloride (anhydrous) and sodium sulfate and the analytical-grade reagent ethanol were obtained from Merck, India, and the HPLC column was procured from Agilent Technologies Pvt. Ltd., India. Water was purified with a Millipore Direct Q purification system (Lab Link, Germany).

### 2.5. Monitoring of dermal exposure

Trained staff was assigned for collecting the samples through patch dosimeters, wipes, and hand-washing methods from the farmworkers to measure the exposure from dermal washings and by following the standard operating procedures (9, 10, 18). The farmworkers were instructed to wash their hands with water before starting work to eliminate possible background contamination, while hand-wash samples were collected at the end of the shift after handling the pesticides. Each farmworker was instructed to rinse one hand at a time for at least 30 s in a Ziploc bag containing 200 ml of ethanol (70% v/v). Surgical cotton-gauze swabs (1 mm thickness, 100 cm^2^ surface area) lined with an impermeable material (aluminum foil) to prevent collected residues from penetrating through the swabs and into the skin and/or clothing were used as a patch sampler. Ten such patch samplers were taped onto the clothing worn by each farmworker (external patch) and applied to various locations on exposed dermal regions of the inner clothing under the PPE (internal patch). A skin wipe technique using a cotton surgical gauze swab moistened with 2 ml of 70% ethanol was used as a wipe sampler to assess the dermal penetration of pesticide residues on exposed dermal regions of the face/forehead and neck at the end of the work shift. At the end of sampling, dermal washing samples (patch, wipe, and hand-wash) were collected in Ziploc bags, labeled, chilled with ice packs, transported from the field to the laboratory, and stored at −20°C (deep-freezer HF 500 CHP; Carrier, USA) to complete the extraction within 7 days of their collection.

The collected hand-wash samples were filtered through Whatman filter paper number 42 and then passed through anhydrous sodium sulfate three times. The filtrate was then fully dried at 30°C and 80 rpm using a rotary evaporator procured from Aditya Scientific, India, and the reconstitution was done using 1 ml of acetonitrile. The ultra-sonication (Ultrasonic Cleaner, Equitron, India) of patch and wipe samples was done for 15 min using 20 ml of methanol, which was further dried using a Turbo-Vap (LV concentrator, Caliper Life Sciences, India) at 30°C and 15 psi under a gentle stream of nitrogen, and the extracted residues were reconstituted using 1 ml of methanol. Both extracts were then filtered using 0.22 μm polytetrafluoroethylene (PTFE) syringe filters (Nupore Filtration Systems, India) into auto-sampler vials (1.5 ml) and stored at −80°C (ultra-low temperature freezer, Haier, China) until analyzed.

### 2.6. Health risk assessment

#### 2.6.1. Non-carcinogenic analysis

The non-cancer health risk of dermal exposure to pesticides among the farmworkers was calculated (19). The average daily doses (ADD, mg kg^−1^ day^−1^) for each pesticide residue were calculated using Equation 1.


(1) $$\begin{array}{l} {\text{ADD}}_{\text{dermal}}\\ =\left(\frac{{\text{C}}_{\text{dermal washings}}\times \text{SA}\times \text{PC}\times \text{ET}\times \text{EF}\times \text{ED}\times \text{CF}}{\text{BW}\times \text{AT}}\right) \end{array}$$

where C_dermal washings_ is the average concentration of pesticide residues detected in dermal washings (hand-wash/patch/wipe samples) (mg); SA is the skin surface area available for contact (cm^2^); PC is the dermal permeation constant of each pesticide residue (cm h^−1^); ET is the daily exposure time (h day^−1^); EF is the exposure frequency (days year^−1^); CF is the conversion factor (0.001 L cm^−3^); ED is the exposure duration (years); AT is the average lifetime exposure (days); and BW is the average body weight (kg). The parameters of ET, EF, ED, BW, and AT were obtained from the results of the questionnaire (Table 1).

**Table 1:** Characteristics of farmworkers (*n* = 120).

**Parameters**	***n* (%)**	**Chi score**	***p*-value**
*Gender*		3.471	0.176
Men (farmmen)	74 (62)		
Women (farmwomen)	46 (38)		
*Education status*		57.041	0.001^**^
Illiterate	31 (20)		
Read and write	58 (10)		
Primary (1st–5th)	12 (13)		
Secondary (6th−12th)	15 (16)		
Graduate	4 (3)		
Daily exposure time (ET)	3 h day^−1^		
Exposure frequency (EF)	154 days year^−1^		
Average extent of land holdings	3.88 acres		
Exposure duration (ED)	mean 18.01 years		
Average body weight (BW)	60 kg		
Average lifetime exposure (AT)	36 years		
*Pesticides exposure history*		20.410	0.026^*^
1–15 years	33 (21)		
15–22 years	45 (19)		
>22 years	42 (22)		
*Type of occupation*		1.880	0.930
Agriculture	38 (23)		
Tenant cultivation	32 (24)		
Agriculture labor	43 (25)		
Other labor	7 (6)		
*Personal habits/diet*			
Non-vegetarians	118 (98)	5.631	0.002^**^
Lacto-ovo-vegetarians	2 (2)		
Smoking cigarettes or *beedis*^#^	78 (65)	3.616	0.164
Consuming alcohol^#^	62 (26)	7.793	0.020^*^
*Use of personal protective equipment (PPE)*		5.668	0.001^**^
Yes	0		
No	119 (99)		
Any other (handkerchief/towel)	1 (1)		
*Mode of mixing of pesticides*		2.264	0.322
Bare handed	106 (88)		
With gloves on	0 (0)		
With the aid of wooden stick or metal rod	14 (15)		
*Pesticides storage practices*		1.394	0.845
In the farm in a separate shed	78 (65)		
In the house in a separate room	29 (27)		
In the house along with other items	13 (14)		
*Disposal of the empty containers of pesticides*		1.965	0.568
In the agricultural fields	47 (28)		
In the canal/passage of the agricultural fields	16 (16)		
In the open/barren fields	19 (29)		
In the dumping ground where the waste material is dumped	17 (30)		
Sell as scrap	21 (31)		

^#^Multiple responses allowed. Statistical significance was considered at ^*^p < 0.05 and ^**^p < 0.01.

The dermal permeation constant (PC) of each pesticide residue was calculated using Equation 2 (20):


(2) $$\begin{array}{l} \text{PC}=10^{\left(-0.280+0.66\text{log Kow}-0.0056\text{MW}\right)} \end{array}$$

where Kow is the octanol-water partition coefficient of each pesticide residue and MW is the molecular weight of each pesticide residue (Supplementary file 2, Table S1).

The non-cancer risk of pesticide residues *via* dermal exposure was represented as HQ, which was calculated using Equation 3:


(3) $$\begin{array}{l} \text{HQ}=\frac{{\text{ADD}}_{\text{dermal}}}{\text{ARfD}} \end{array}$$

where HQ is the hazard quotient of exposure, and ARfD (mg kg^−1^ day^−1^) represents the daily maximum permissible level of pesticide residues, including the reference dose for ingestion and dermal contact (21, 22). Out of 10 pesticide residues detected, 9 (except phenthoate) have ARfD values, and hence, the HQ was calculated only for 9 pesticide residues (Supplementary file 2, Table S1).

#### 2.6.2. Hazard index for multiple pesticide residues

The total potential non-carcinogenic risks caused by occupational dermal exposure to a mixture of pesticides were estimated in terms of the Hazard Index (HI). The HI for several pesticide residues was calculated using Equation 4 (23):


(4) $$\begin{array}{l} \text{HI}=\text{ΣHQ}={\text{HQ}}_{\text{acephate}}+{\text{HQ}}_{\text{monocrotophos}}+{\text{HQ}}_{\text{quinalphos}}\\ \;+{\text{HQ}}_{\text{profenofos}}+{\text{HQ}}_{\text{chlorpyrifos}}+{\text{HQ}}_{\text{phorate}}+{\text{HQ}}_{\text{dimethoate}}\\ \;+{\text{HQ}}_{\text{emamectin benzoate}}+{\text{HQ}}_{\text{imidacloprid}} \end{array}$$

The risks of pesticide residues measured will be relatively higher when HI is > 1, while the risks may be considered negligible when the value of HI is < 1.

### 2.7. Margin of safety

The MOS, a risk indicator, was measured as previously reported (24, 25) for each commonly/predominantly used pesticide by randomly selecting 10 farmworkers not using PPE or following any specified GAPs, using Equation 5:


(5) $$\begin{array}{l} \text{MOS}=\left[\text{AOEL or NOAEL}\times \text{BW}/\left(\sum \text{DE}\times 0.11\right)\right] \end{array}$$

Values of MOS ≥ 1 represent safe working conditions, while values of MOS of < 1 indicate unsafe working conditions. When Acceptable Operator Exposure Levels (AOELs) were not available, No Observed Adverse Effect Levels (NOAELs) were used based on an average body weight (BW) of 60 kg. The absorption factor (AF) value was taken as 0.11, corresponding to 10% percutaneous absorption plus 1% to account for the inhalation rate. DE (dermal exposure) is the summation of dermal exposure obtained from the levels of pesticide residues (μg) obtained from the analysis of dermal washing samples (patch, wipe, and hand-wash) collected by a farmworker for all the agricultural tasks (mixing/loading/application) that were completed for that shift/day.

### 2.8. Quality control

**Figure 1:**
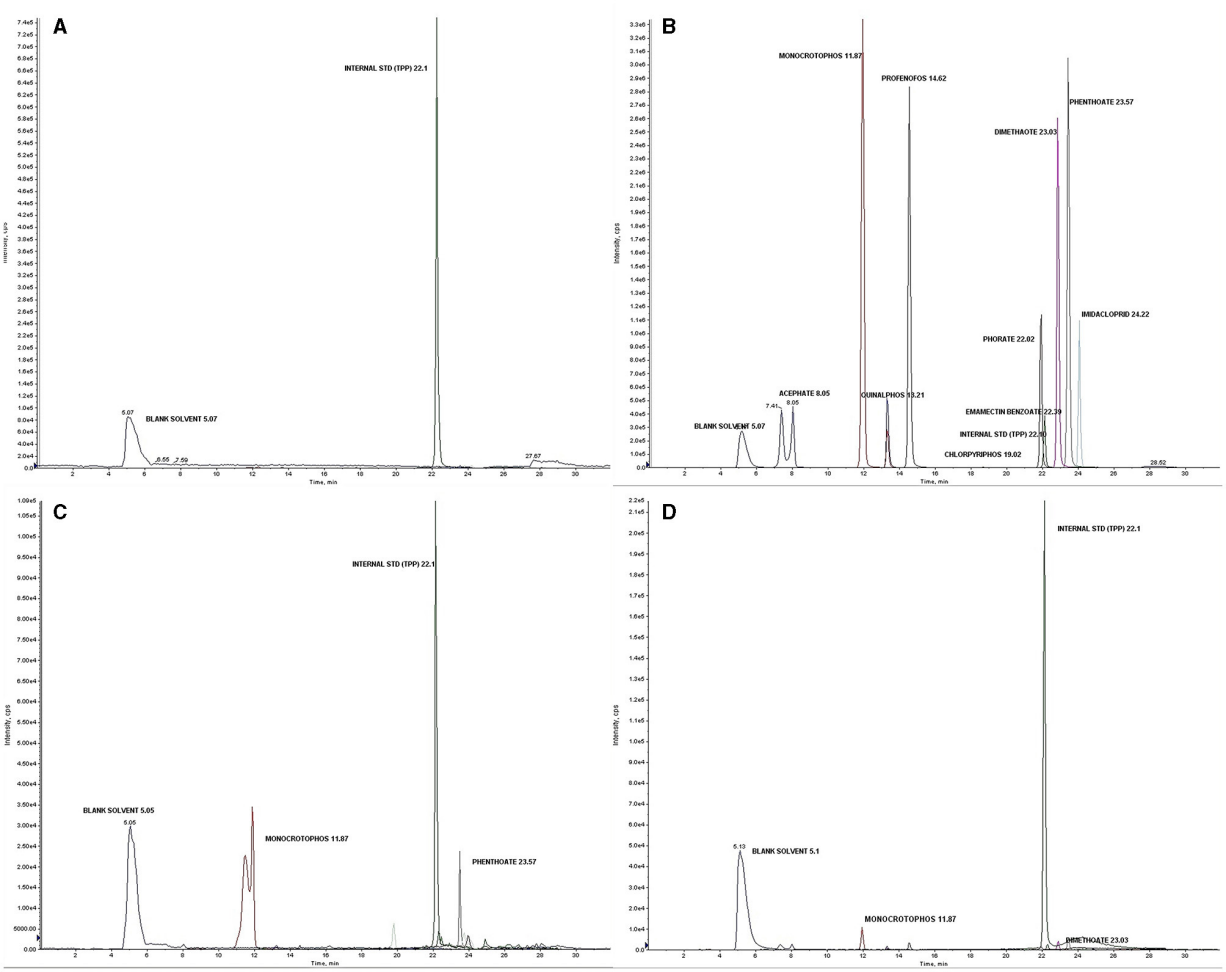
**(A)** Blank solvent chromatogram; **(B)** Total ion chromatogram (TIC) showing analyzed pesticide residues with an internal standard (Triphenyl phosphate) at a concentration of 1,000 ng ml^−1^; **(C)** Chromatogram of the detected pesticides in a real sample of a farmworker not using PPE; **(D)** Chromatogram of the detected pesticides in a real sample of farmworker after using PPE.

Liquid chromatography with tandem mass spectrometry (LC-MS/MS) instrument conditions used for the estimation of pesticide residues are tabulated (Table 2). The standardized method used for the quantitative analyses and qualitative confirmation of pesticide residues in dermal washing samples (patch/wipe/hand-wash) was modified and validated in-house prior to the real sample analyses (26–28). The mass parameters (MS-MS) have been optimized by multiple reaction monitoring (MRM) modes (Supplementary file 2, Table S2). The experimental limit of detection (LOD) and the limit of quantitation (LOQ) were determined for each analyte at a signal-to-noise ratio (S/N) of 3:1 and 10:1, respectively. The blank run between the two samples did not show any carryover from the previous sample run. Consistent retention over 10 runs was observed with a retention time variation of ±0.2 min and < 3% relative standard deviation (RSD) of peak areas (Figure 1). Calibration curves were found to be linear with a correlation coefficient (R) ranging from 0.998 to 0.999. The precision was determined as an RSD in terms of repeatability (intra-day) and reproducibility (inter-day) at three different concentrations of 1 or 5 (LOQ of analyte), 50, and 500 ng ml^−1^. Quality control parameters of the LC-MS/MS method for the determination of pesticide residues in hand-wash (Supplementary file 2, Table S3) and patch dosimeter and wipe samples (Supplementary file 2, Table S4) were determined.

**Table 2:** Liquid chromatography with tandem mass spectrometry (LC-MS/MS) condition.

Instrument	Liquid chromatography system (Shimadzu LC 20AD) equipped with a mass spectrometer (Applied Biosystems MDS Sciex 4000-Q TRAP triple quadruple) and auto-sampler (SIL-HTC model)
Software	Analyst Software (version 4.1.2)
Column	Zorbax SB-C18 HPLC column (internal diameter of 4.6, 250 mm length, and 5 μm particle size)
Oven temperature	40°C (25°C minimum; 85°C maximum)
Mobile phases	Mobile phase A: milli-Q water containing 0.1% formic acid; Mobile phase B: methanol with 0.1% formic acid
Gradient program	Pump B 10% from 0.01 min to 20 min; 98% till 25 min, 10% till 32min
Flow rate	800 μl min^−1^
Injection volume	35 μl
Mass spectrometry conditions	Multiple reaction monitoring (MRM) positive turbo Electrospray Ionization (ESI) mode with high resolution
Ion spray voltage (IS)	5,500 eV
Interface heater temperature	500°C
Total run time	32 min

### 2.9. Statistical analysis

The data were subjected to statistical analysis using the Statistical Package for Social Sciences (SPSS) version 28.0.0.0 21 (29) and were expressed as mean (standard deviation), frequency, and percentage. The associations between demographic information and various knowledge, attitude, and practice parameters were assessed using the Pearson chi-square analysis. In addition, a paired *t*-test or Wilcoxon signed-rank test was performed to assess the significance between exposure levels of farmworkers before and after using PPE for different exposed dermal regions based on the assumption of normality. The associations were determined at confidence intervals (CIs) of 95%, and the statistical significance of *p*-values of < 0.05 and < 0.01 was considered.

## 3. Results

### 3.1. Characteristics of farmworkers

The details of the self-reported information provided by the farmworkers studied have been reported earlier (30, 31), while the self-reported information collected using a pre-tested questionnaire and other field observations from selected farmworkers (*n* = 120) are presented (Table 1). Furthermore, it was observed that 94% of the farmworkers were using nearly 10-year-old defective backpack sprays with leakages from nozzles and pipes, due to which there is likely exposure of the farmworkers' body parts to the droplets coming out of the nozzles of the knapsack sprayer. Furthermore, they were also observed to be applying the pesticide formulations with the lance kept ~30 cm above the top of the crop in front of them and swinging it sideways, which results in the formation of a spray aerosol that could obstruct the way forward. In addition, the majority of the farmworkers (98%) rely on the retailers for the type/quantity of pesticides to be sprayed onto the crops cultivated by them as they were unaware of the exact names or quantities of pesticide formulations to be used. This, however, depends on or varies as per the crop that is being cultivated, the intensity of the pest infestation, and their need for treatment. Therefore, details of the types of pesticides used by farmworkers were obtained from neighboring retailers and the *Krishi Vigyan Kendras* of agricultural extension centers located in the identified villages of each area studied. It was found that organophosphate pesticides (acephate, profenofos, chlorpyrifos, monocrotophos, phorate, quinalphos, and phenthoate) were most commonly and frequently used by them, followed by other classes of pesticides (neonicotinoids: imidacloprid; carbamates: mancozeb, carbosulfan, and carbendazim; synthetic pyrethroids: cypermethrin, lambda-cyhalothrin, and deltamethrin).

The meteorological conditions recorded indicated high temperatures ranging between 27.8°C and 41°C (mean 35.28°C), relative humidity ranging from 7.2 to 65.4% (mean 34.36%), and an average southwest wind velocity of 8.91 km h^−1^ during their involvement in the farming activities. It was also observed that most of them were spraying the pesticide formulations against the wind's direction. Furthermore, 99% of farmworkers observed to have not used any PPE of their own while handling the pesticides; however, the reasons indicated for not using the same varied as some (44%) stated inconvenience, while it was inaccessible for 51%, and others (5%) stated the feeling of suffocation. It was further found that none of the participants had received any official professional training on pesticide handling or GAPs. In addition, the majority of them also explained self-reported associated morbidity symptoms of weakness, itching of the skin, red eyes/burning sensation in the eyes, diarrhea, headache, nausea, vomiting, abdominal cramps, etc., immediately after spraying the pesticides.

### 3.2. Dermal exposure to pesticides

#### 3.2.1. Pesticide residues in hand-wash samples

The analysis done for the hand-washing methods among the farmworkers who have not used any PPE (*n* = 120) showed 10 pesticide residues: acephate, monocrotophos, quinalphos, profenofos, chlorpyrifos, phorate, dimethoate, emamectin benzoate, imidacloprid, and phenthoate. Of the 10 pesticide residues detected, quinalphos (*n* = 90), monocrotophos (*n* = 88), and profenofos (*n* = 81) were found among a larger number of farmworkers, while imidacloprid was detected at the highest mean ± SD concentration (19.27 ± 46.96 μg ml^−1^) when compared to the other residues. The concentration of the detected pesticide residues ranged from 0.000 to 246 μg ml^−1^ (Tables 3, 4).

**Table 3:** Pesticide residues detected in hand-wash samples among farmworkers not using PPE (*n* = 60) and using commercially available PPE (*n* = 60).

**Analyte**	**Without using PPE (*****n*** **=** **60)**			**After using commercially available PPE (*****n*** **=** **60)**			***p*-value**
	**Detection frequency *n* (%)**	**Mean ±standard deviation (μg ml^−1^)**	**Range**	**Detection frequency *n* (%)**	**Mean ±standard deviation (μg ml^−1^)**	**Range**	
Acephate	29 (4)	7.204 ± 29.361	0.00–188.00	22 (40)	0.684 ± 1.422	0.00–8.40	0.326
Monocrotophos	49 (82)	13.381 ± 21.391	0.00–82.00	55 (92)	1.401 ± 2.522	0.00–11.64	0.000^**^
Quinalphos	53 (88)	9.031 ± 17.612	0.00–75.00	17 (21)	0.415 ± 1.301	0.00–5.78	0.000^**^
Profenofos	48 (80)	2.601 ± 9.082	0.00–44.00	26 (41)	0.531 ± 1.621	0.00–9.30	0.008^**^
Chlorpyrifos	46 (77)	4.432 ± 25.581	0.00–195.00	18 (42)	0.073 ± 0.204	0.00–0.98	0.001^**^
Phorate	13 (38)	0.032 ± 0.221	0.00–1.73	5 (11)	0.024 ± 0.163	0.00–1.23	0.002^**^
Dimethoate	6 (13)	0.002 ± 0.013	0.00–0.05	ND	-	-	0.968
Emamectin benzoate	12 (35)	3.255 ± 15.912	0.00–120.60	20 (43)	0.802 ± 2.215	0.00–13.74	0.605
Imidacloprid	38 (63)	19.271 ± 46.961	0.00–246.00	16 (24)	1.334 ± 3.472	0.00–16.00	0.028^*^
Phenthoate	15 (20)	0.164 ± 1.073	0.00–8.26	ND	-	-	0.001^**^

Statistical significant differences was considered at ^*^p < 0.05; ^**^p < 0.01.

**Table 4:** Pesticide residues detected in hand-wash samples among farmworkers not using any PPE (*n* = 60) and using PPE prepared using available resources (*n* = 60).

**Analyte**	**Without using PPE (*****n*** **=** **60)**			**After using PPE prepared using available resources (*****n*** **=** **60)**			***p*-value**
	**Detection frequency *n* (%)**	**Mean ±standard deviation (μg ml^−1^)**	**Range**	**Detection frequency *n* (%)**	**Mean ±standard deviation (μg ml^−1^)**	**Range**	
**Acephate**	26 (41)	3.808 ± 25.561	0.00–198.00	6 (13)	0.001 ± 0.003	0.00–0.02	0.000^**^
**Monocrotophos**	39 (65)	10.591 ± 21.472	0.00–77.40	7 (15)	0.002 ± 0.015	0.00–0.12	0.000^**^
**Quinalphos**	37 (62)	2.101 ± 6.445	0.00–36.20	11 (31)	0.017 ± 0.083	0.00–0.50	0.000^**^
**Profenofos**	33 (44)	0.532 ± 1.583	0.00–9.22	37 (62)	0.012 ± 0.031	0.00–0.20	0.000^**^
**Chlorpyrifos**	32 (45)	0.865 ± 3.891	0.00–23.40	10 (33)	0.000 ± 0.000	0.00–0.00	0.000^**^
**Phorate**	9 (32)	0.001 ± 0.001	0.00–0.02	ND	-	-	0.008^**^
**Dimethoate**	5 (11)	0.014 ± 0.071	0.00–0.43	3 (6)	0.001 ± 0.016	0.00–0.04	0.327
**Emamectin benzoate**	13 (38)	0.381 ± 1.862	0.00–13.80	4 (8)	0.003 ± 0.000	0.00–0.12	0.004^**^
**Imidacloprid**	37 (62)	8.889 ± 16.261	0.00–59.80	15 (20)	0.248 ± 0.015	0.00–11.44	0.000^**^
**Phenthoate**	3 (6)	0.001 ± 0.011	0.00–0.05	ND	-	-	0.109

Statistical significant differences was considered at ^*^p < 0.05; ^**^p < 0.01.

Furthermore, it was observed that there was a reduction both in the concentrations and the number of pesticide residues detected in the hand-wash samples of the farmworkers who have used commercially available PPE and PPE prepared using available resources provided to them when compared with those who have not used PPE. Residues of eight pesticides (acephate, monocrotophos, quinalphos, profenofos, chlorpyrifos, phorate, emamectin benzoate, and imidacloprid) were detected among those who had commercially available PPE provided to them (*n* = 60), in the range of 0.000 to 16 μg ml^−1^. A significant difference (*p* < 0.01) was found for seven pesticide residues (monocrotophos, quinalphos, profenofos, chlorpyrifos, phorate, imidacloprid, and phenthoate) when no PPE was used vs. after the use of commercially available PPE (Table 3).

While residues of eight pesticides (acephate, monocrotophos, quinalphos, profenofos, chlorpyrifos, dimethoate, emamectin benzoate, and imidacloprid) were detected among the samples collected from those farmworkers provided with PPE prepared using available resources (*n* = 60) in concentrations ranging from 0.000 to 11.44 μg ml^−1^, there was a significant difference (*p* < 0.01) in the residual levels when they did not use PPE and after using PPE prepared using available resources (Table 4).

#### 3.2.2. Pesticide residues in patch samples

Residues of ten pesticides (acephate, monocrotophos, quinalphos, profenofos, chlorpyrifos, phorate, dimethoate, emamectin benzoate, imidacloprid, and phenthoate) in the range of 0.000 to 198.33 ng cm^−2^ were detected in patch samples from farmworkers not using PPE (*n* = 120). Of them, monocrotophos (*n* = 90) and quinalphos (*n* = 87) were detected abundantly, with monocrotophos being detected at a higher mean ± SD concentration (12.23 ± 36.96 ng cm^−2^) (Tables 5, 6).

**Table 5:** Pesticide residues detected in patch samples among farmworkers not using any PPE (*n* = 60) and using commercially available PPE (*n* = 60).

**Analyte**	**Without using PPE (*****n*** **=** **60)**			**After using commercially available PPE (*****n*** **=** **60)**			***p*-value**
	**Detection frequency *n* (%)**	**Mean ±standard deviation (ng cm^−2^)**	**Range**	**Detection frequency *n* (%)**	**Mean ±standard deviation (ng cm^−2^)**	**Range**	
Acephate	27 (46)	7.823 ± 33.099	0.00–188.33	17 (21)	0.311 ± 0.937	0.00–5.37	0.047^*^
Monocrotophos	50 (83)	8.392 ± 18.673	0.00–98.67	55 (92)	1.260 ± 1.337	0.00–5.58	0.397
Quinalphos	53 (88)	2.960 ± 12.358	0.00–66.83	13 (38)	0.025 ± 0.097	0.00–0.70	0.000^**^
Profenofos	47 (78)	0.193 ± 0.418	0.00–2.55	24 (47)	0.047 ± 0.132	0.00–0.66	0.000^**^
Chlorpyrifos	46 (77)	0.297 ± 0.657	0.00–3.28	16 (24)	0.057 ± 0.160	0.00–0.82	0.001^**^
Phorate	26 (41)	0.039 ± 0.088	0.00–0.41	3 (6)	0.000 ± 0.000	0.00–0.00	0.000^**^
Dimethoate	24 (47)	0.119 ± 0.336	0.00–1.90	ND	-	-	0.000^**^
Emamectin benzoate	13 (38)	0.259 ± 0.853	0.00–76.50	19 (23)	0.049 ± 0.159	0.00–0.90	0.264
Imidacloprid	33 (44)	3.293 ± 11.744	0.00–5.58	18 (42)	0.280 ± 1.136	0.00–6.57	0.005
Phenthoate	30 (17)	0.120 ± 0.274	0.00–1.36	ND	-	-	0.000^**^

Statistical significant differences was considered at ^*^p < 0.05; ^**^p < 0.01.

**Table 6:** Pesticide residues detected in patch samples among farmworkers not using any PPE (*n* = 60) and using PPE prepared using available resources (*n* = 60).

**Analyte**	**Without using PPE (*****n*** **=** **60)**			**After using PPE prepared using available resources (*****n*** **=** **60)**			***p*-value**
	**Detection frequency *n* (%)**	**Mean ±standard deviation (ng cm^−2^)**	**Range**	**Detection frequency *n* (%)**	**Mean ±standard deviation (ng cm^−2^)**	**Range**	
Acephate	18 (42)	3.332 ± 23.869	0.00–185.00	ND	-	-	0.000^**^
Monocrotophos	40 (67)	12.231 ± 36.958	0.00–198.33	2 (3)	0.005 ± 0.039	0.00–0.30	0.000^**^
Quinalphos	34 (48)	1.432 ± 6.901	0.00–40.17	6 (13)	0.000 ± 0.000	0.00–0.00	0.000^**^
Profenofos	31 (26)	0.037 ± 0.090	0.00–0.42	10 (33)	0.002 ± 0.004	0.00–0.01	0.000^**^
Chlorpyrifos	39 (65)	1.097 ± 7.782	0.00–60.33	29 (4)	0.000 ± 0.001	0.00–0.01	0.000^**^
Phorate	11 (31)	0.021 ± 0.110	0.00–0.83	ND	-	-	0.003^**^
Dimethoate	5 (11)	0.027 ± 0.147	0.00–1.07	ND	-	-	0.043^*^
Emamectin benzoate	13 (38)	0.065 ± 0.281	0.00–2.05	3 (6)	0.014 ± 0.086	0.00–0.65	0.084
Imidacloprid	18 (42)	0.740 ± 3.539	0.00–26.83	20 (43)	0.095 ± 0.290	0.00–1.82	0.321
Phenthoate	5 (11)	0.011 ± 0.056	0.00–0.43	ND	-	-	0.043^*^

Statistical significant differences was considered at ^*^p < 0.05; ^**^p < 0.01.

Interestingly, the mean concentration values of the pesticide residues detected in the patch samples among farmworkers using commercially available PPE were low when compared to those not using PPE. Residues of eight pesticides (acephate, monocrotophos, quinalphos, profenofos, chlorpyrifos, phorate, emamectin benzoate, and imidacloprid) were only detected when they used commercially available PPE provided to them (*n* = 60) in the range from 0.000 to 6.57 ng cm^−2^. Data analysis also revealed a significant difference (*p* < 0.01) in the exposure levels among the farmworkers not using PPE vs. after their use of commercially available PPE for seven pesticide residues (acephate, quinalphos, profenofos, chlorpyrifos, phorate, dimethoate, and phenthoate) (Table 5).

There was a relatively lower number of pesticide residues detected with lower mean concentration levels in the patch samples when the farmworkers used PPE prepared using available resources when compared to those not using PPE. Residues of six pesticides (monocrotophos, quinalphos, profenofos, chlorpyrifos, emamectin benzoate, and imidacloprid) were detected in the patch samples collected from the farmworkers who have used PPE prepared using available resources provided to them (*n* = 60), and their concentration values ranged from 0.000 to 1.82 ng cm^−2^, showing a significant difference (*p* < 0.01) for eight pesticide residues detected (acephate, monocrotophos, quinalphos, profenofos, chlorpyrifos, phorate, dimethoate, and phenthoate) as compared to those not using PPE (Table 6).

#### 3.2.3. Pesticide residues in wipe samples

With regard to the wipe samples collected from the farmworkers who are not using PPE (*n* = 120), 10 pesticide residues (acephate, monocrotophos, quinalphos, profenofos, chlorpyrifos, phorate, dimethoate, emamectin benzoate, imidacloprid, and phenthoate) were detected, of which, monocrotophos (*n* = 85), chlorpyrifos (*n* = 80), profenofos (*n* = 79), and quinalphos (*n* = 75) were detected in a greater number of farmworkers, with monocrotophos being detected at a higher mean ± SD concentration (44.88 ± 152.33 ng cm^−2^) among those who are not using PPE with their concentrations ranging from 0.000 to 1,740 ng cm^−2^ (Tables 7, 8).

**Table 7:** Pesticide residues detected in wipe samples among farmworkers not using any PPE (*n* = 60) and using commercially available PPE (*n* = 60).

**Analyte**	**Without using PPE (*****n*** **=** **60)**			**After using commercially available PPE (*****n*** **=** **60)**			***p*-value**
	**Detection frequency *n* (%)**	**Mean ±standard deviation (ng cm^−2^)**	**Range**	**Detection frequency *n* (%)**	**Mean ±standard deviation (ng cm^−2^)**	**Range**	
Acephate	28 (9)	30.097 ± 224.510	0.00–1,740.00	21 (49)	3.795 ± 10.117	0.00–67.10	0.116
Monocrotophos	50 (83)	14.691 ± 61.097	0.00–459.00	55 (92)	14.631 ± 15.307	0.00–72.90	0.001^**^
Quinalphos	49 (82)	12.593 ± 45.422	0.00–298.00	18 (42)	0.500 ± 1.322	0.00–9.03	0.004^**^
Profenofos	50 (83)	0.140 ± 0.454	0.00–3.17	25 (18)	0.885 ± 1.937	0.00–8.22	0.097
Chlorpyrifos	44 (73)	1.552 ± 3.680	0.00–20.20	17 (21)	1.088 ± 6.468	0.00–49.60	0.001^**^
Phorate	8 (16)	0.038 ± 0.222	0.00–1.68	5 (11)	0.025 ± 0.142	0.00–1.06	0.875
Dimethoate	ND	-	-	ND	-	-	1.000
Emamectin benzoate	13 (38)	0.261 ± 0.907	0.00–5.39	17 (21)	0.581 ± 1.192	0.00–5.31	0.098
Imidacloprid	17 (21)	2.487 ± 10.308	0.00–59.90	22 (40)	6.663 ± 19.456	0.00–114.00	0.104
Phenthoate	8 (16)	0.190 ± 0.751	0.00–4.77	ND	-	-	0.012^*^

Statistical significant differences was considered at ^*^p < 0.05; ^**^p < 0.01.

**Table 8:** Pesticide residues detected in wipe samples among farmworkers not using any PPE (*n* = 60) and using PPE prepared using available resources (*n* = 60).

**Analyte**	**Without using PPE (*****n*** **=** **60)**			**After using PPE prepared using available resources (*****n*** **=** **60)**			***p*-value**
	**Detection frequency *n* (%)**	**Mean ±standard deviation (ng cm^−2^)**	**Range**	**Detection frequency *n* (%)**	**Mean ±standard deviation (ng cm^−2^)**	**Range**	
Acephate	19 (23)	0.746 ± 3.378	0.00–22.00	4 (8)	0.012 ± 0.080	0.00–0.62	0.001^**^
Monocrotophos	35 (58)	44.88 ± 152.329	0.00–856.00	5 (11)	0.003 ± 0.014	0.00–0.11	0.000^**^
Quinalphos	26 (41)	4.112 ± 29.795	0.00–231.00	9 (32)	0.193 ± 1.446	0.00–11.20	0.000^**^
Profenofos	29 (4)	0.393 ± 1.274	0.00–7.54	11 (31)	0.017 ± 0.041	0.00–0.13	0.003^**^
Chlorpyrifos	36 (60)	0.579 ± 1.223	0.00–6.96	28 (9)	0.017 ± 0.036	0.00–0.17	0.000^**^
Phorate	5 (11)	0.029 ± 0.219	0.00–1.70	ND	-	-	0.043^*^
Dimethoate	1 (2)	0.000 ± 0.000	0.00–0.01	2 (3)	0.001 ± 0.009	0.00–0.07	0.593
Emamectin benzoate	9 (32)	0.093 ± 0.319	0.00–1.52	1 (2)	0.001 ± 0.007	0.00–0.05	0.009^**^
Imidacloprid	17 (21)	1.808 ± 9.173	0.00–69.80	18 (42)	0.746 ± 1.695	0.00–8.80	0.957
Phenthoate	1 (2)	0.000 ± 0.001	0.00–0.01	ND	-	-	0.317

Statistical significant differences was considered at ^*^p < 0.05; ^**^p < 0.01.

In contrast, the mean concentration values of the same were low in the wipe samples among those using PPE provided (commercially available/prepared using available resources) when compared to those not using PPE. Residues of eight pesticides (acephate, monocrotophos, quinalphos, profenofos, chlorpyrifos, phorate, emamectin benzoate, and imidacloprid) were detected when they used commercially available PPE (*n* = 60), and their concentration ranged from 0.000 to 114.00 ng cm^−2^, showing a significant difference (*p* < 0.01) in the exposure levels for four pesticide residues (monocrotophos, quinalphos, chlorpyrifos, and phenthoate) when compared to those not using PPE (Table 7).

Furthermore, residues of eight pesticides, acephate, monocrotophos, quinalphos, profenofos, chlorpyrifos, dimethoate, emamectin benzoate, and imidacloprid, were detected in the wipe samples in the concentration ranging from 0.000 to 11.20 ng cm^−2^ among the farmworkers (*n* = 60) when they used PPE prepared using available resources, showing a significant difference (*p* < 0.01) in exposure levels for seven pesticide residues (acephate, monocrotophos, quinalphos, profenofos, chlorpyrifos, phorate, and emamectin benzoate) (Table 8).

#### 3.2.4. Human health risk assessments

The non-cancer risk due to pesticide residues *via* dermal exposure among the farmworkers was determined and revealed that the HQ values of four pesticide residues (monocrotophos, quinalphos, profenofos, and chlorpyrifos) were >1, while they were < 1 for acephate, phorate, dimethoate, emamectin benzoate, and imidacloprid. The order of pesticide residue ranking based on HQ was quinalphos > chlorpyrifos > profenofos > monocrotophos > emamectin benzoate > acephate > phorate > imidacloprid > dimethoate. Furthermore, the cumulative risk evaluated in terms of the HI value for all the detected pesticide residues was found to be >1 (Table 9).

**Table 9:** Non-cancer health risks posed by dermal exposure to pesticides among the farmworkers.

**Pesticides**	**ADD_dermal_**	**HQ**
Acephate	0.0016	0.39
Monocrotophos	0.0037	1.86
Quinalphos	0.5149	1029.82
Profenofos	0.0349	17.56
Chlorpyrifos	0.3359	33.59
Phorate	0.0002	0.08
Dimethoate	0.0000	0.02
Emamectin benzoate	0.0005	0.66
Imidacloprid	0.0052	0.04
HI		**1084.00**

ADD, Average daily doses (mg kg^−1^ day^−1^); HQ, hazard quotient; HI, hazard index.

#### 3.2.5. Margin of safety

**Figure 2:**
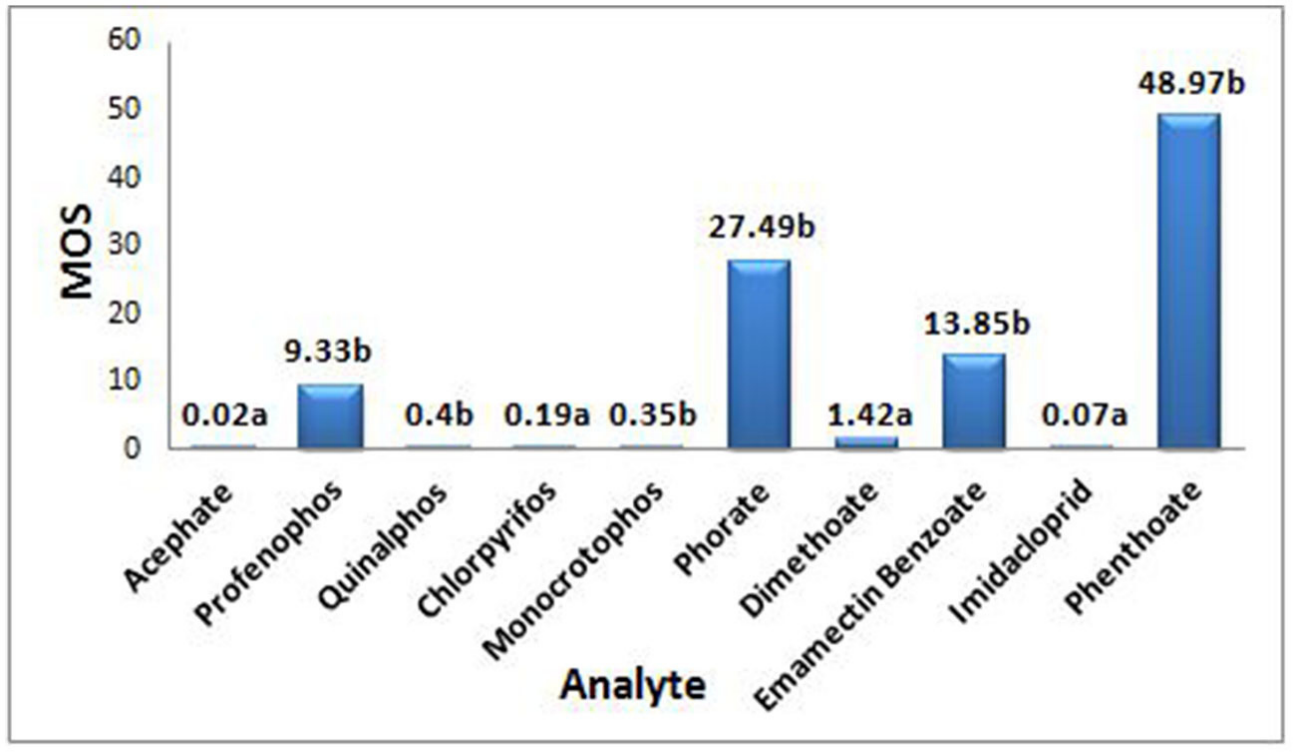
MOS is Margin of safety; a depending on Acceptable Operator Exposure Level (AOEL), AOEL (mg ai/kg-bw/day) values for acephate - 0.0008, chlorpyrifos - 0.001, dimethoate - 0.001, imidacloprid - 0.008, b depending on No Observed Adverse Effect Level (NOAEL), NOAEL (mg ai/kg-bw/day) values for profenophos - 1.0, quinalphos - 0.05, monocrotophos - 0.005, phorate - 0.05, Emamectin benzote - 0.6, phenthoate - 0.1.

The MOS was calculated for each pesticide residue based on the AOEL or NOAEL values of pesticides (as the risk assessment is highly dependent on the toxicological properties of individual active ingredients) so as to determine the safety of a pesticide for its use based on a single instance. In the present study, the obtained MOS value for acephate, quinalphos, chlorpyrifos, monocrotophos, and imidacloprid was found to be lower than 1 (ranged between 0.02 and 0.4), indicating unsafe handling practices followed for the one-time exposure under the prevailing conditions, while, in contrast, it was >1 for pesticides such as profenofos, phorate, dimethoate, emamectin benzoate, and phenthoate, showing that the spraying done under the then specified conditions was observed as safe in terms of their respective acceptable daily exposure limits (Figure 2).

**Figure 3:**
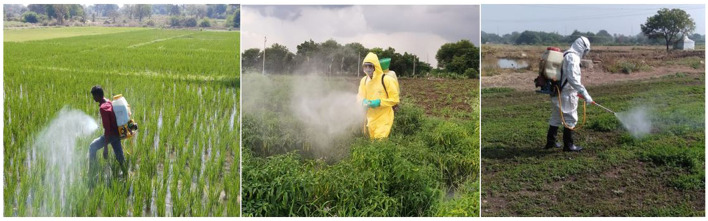
Farmworkers involved in farming activities without following safety protocol and after using provided PPE.

**Figure 4:**
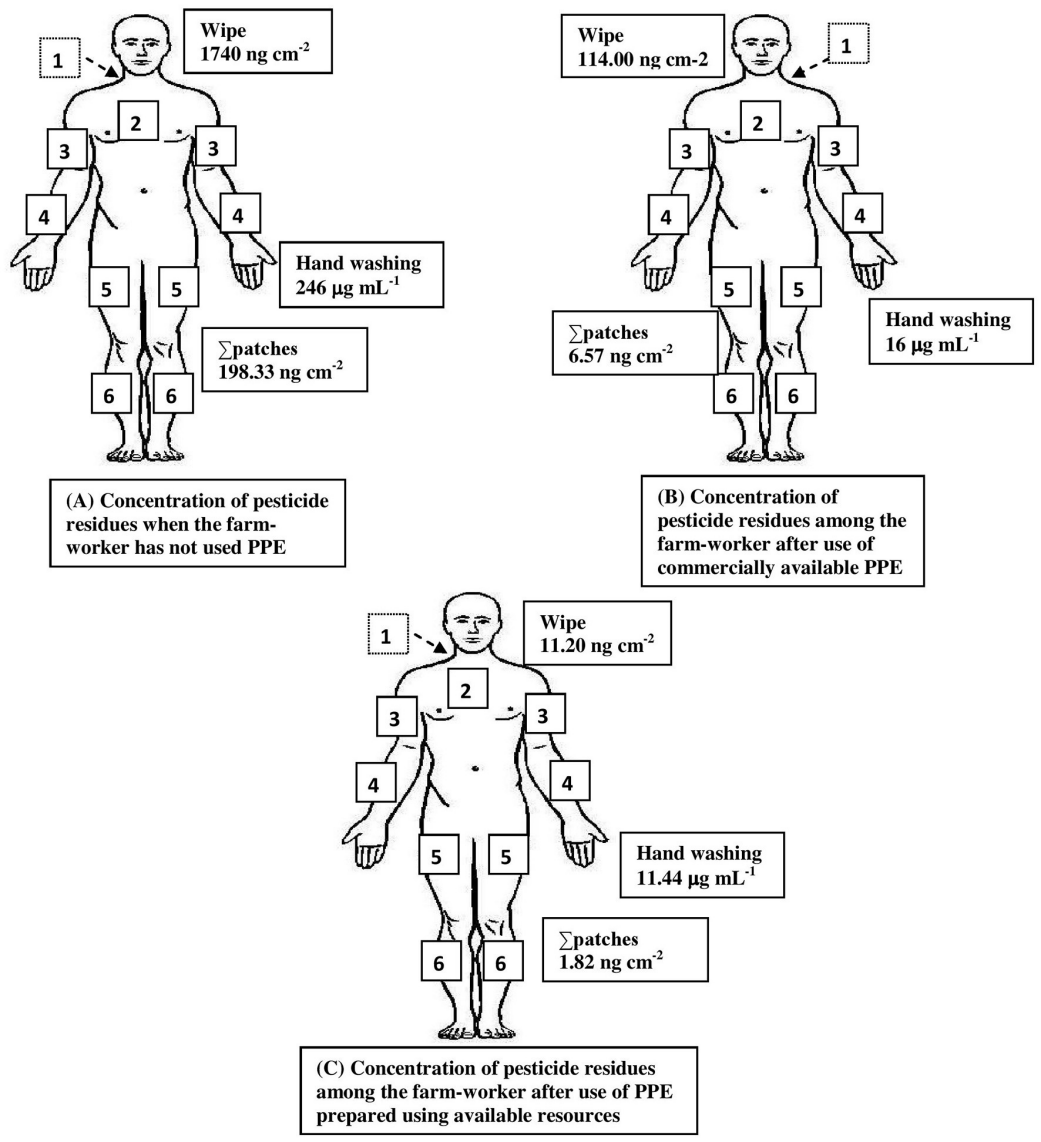
Summary of pesticide exposure among farmworkers not using PPE **(A)** and minimization of exposure after use of commercially available PPE **(B)** and PPE prepared using available resources **(C)**. The numbers represent the location of patches on the following body regions: 1. On the back between shoulder blades; 2. Over sternum; 3. the upper surface of right/left forearm; 4. At right angle across the body, midway between elbow and wrist forearm; 5. Front of right/left leg and mid-thigh; and 6. Front of right/left leg and above ankle-below knee. Values indicate the maximum concentration obtained from the analysis of hand-washing techniques of both hands, a wipe sample of the exposed face and neck region, and patches from other exposed body regions.

On the whole, the results of the analysis of the dermal washing (patch, wipe, and hand-wash) samples revealed that the highest concentration of pesticide residues was detected in the hand-wash samples, followed by wipe samples, implying that the distribution of contamination was particularly high in the hand region, followed by the face/neck among the studied farmworkers who have not used PPE. Furthermore, it was found that the protection offered against dimethoate and phenthoate exposure was absolute (100%) among those who have used commercially available PPE, while it was only for phenthoate among those using the PPE prepared using the available resources provided to them (Figures 3, 4).

## 4. Discussion

Though dermal exposure to pesticides is a major route of occupational exposure for pesticide applicators, there is a paucity of the availability of both the data and methods to accurately estimate the associated risks (32, 33). Additionally, for the past six decades, academics is emphasizing that the use of PPE and the adoption of GAPs would reduce the risk due to exposure (34, 35). The current epidemiological study is the first of its kind to assess dermal exposure to pesticides among Indian farmers in a real-time field setting using patch dosimetry, hand-washing, and wipe techniques. It also highlights the need of using PPE in minimizing exposure.

In the present investigation, the results of the data obtained based on the self-reported information among farmworkers identified several potential risk factors, including a lack of technical knowledge and awareness of pesticide toxicity, inappropriate clothing, bare-hand mixing of pesticide formulations, spraying using defective equipment, working in erratic tropical climatic conditions without following the wind directions, inadequately observed personal protection during the use of pesticides, and their reluctance/ignorance to obey GAPs. These risk factors might increase the likelihood of exposure at higher levels and also contribute to adverse health effects, which is consistent with previous study findings in other developing nations (36, 37). Moreover, the use of potentially cancer-causing pesticides by farmworkers, including those classified as moderately (class II) and extremely/highly hazardous (class Ia/Ib), in combination with repeated exposure to pesticides that are prohibited elsewhere, is also a cause for concern (38). Additionally, the majority (99%) of the farmworkers in the present investigation did not use PPE while engaged in various farming activities, allowing pesticide entry (27, 39). Besides that, the farmworkers' self-reported morbidity symptoms were in agreement with those reported by other pesticide applicators from different regions (2, 40).

It is further evident from earlier study results that the pulse dosimeter, wipe, and hand-washing methods are the most frequently used to measure dermal exposure to pesticides because of their obvious advantages of cost-effectiveness and simplicity (41, 42). In the present study, a simultaneous multi-residue method was developed and validated to estimate 10 pesticide residues that were commonly and predominantly used in the studied areas. The developed method was found to be satisfactory in terms of recovery, correlation, and precision obtained.

Study findings revealed that hand contamination represents the major contribution to dermal exposure among the farmworkers when PPE was not used; on the contrary, hand-wash samples of farmworkers showed minimum exposure after using the PPE provided to them. It was found that exposure through hand contamination accounted for >71% of the total dermal exposure, which could have been due to the bare hands mixing/loading of the concentrated pesticide liquid formulations to the spray equipment/tanks for spraying purposes. In addition, the need for contact with the spray equipment/tanks was observed as frequent during their participation in farming activities, followed by the likely deposition of residual droplets on the exposed dermal regions, especially the hands. Study results are in agreement with earlier studies conducted elsewhere, indicating that hands account for a substantial portion of total dermal exposure (42–45). It was also reported in other studies that the exposure through hands while using liquid formulations was observed to be 22–62 times greater than that of the solid formulations, highlighting the importance of using PPE resulting in negligible contamination through hands, which was further in accordance with the present study findings (24, 46).

The pesticide levels analyzed using the patch dosimeter and skin-wipe technique also revealed that the exposure through the face/neck regions was another important source of dermal exposure and a source of concern among most of the farmworkers as they were frequently using their contaminated bare hands for wiping their sweat on their faces in the prevailing hot and humid climatic conditions. Contrarily, among the various types of exposure, the head and face were found to be one of the major contributors to dermal exposure among the applicators, despite being only occasionally reported as an important route of exposure (47). Earlier studies conducted elsewhere have also reported residues in similar samples (48, 49). In the present investigation, a significant reduction in the pesticide residual concentration (*p* < 0.01) in patch and wipe samples collected from the farmworkers was observed when they used PPE. Studies conducted also observed similar findings showing the importance of the use of PPE in providing significant protection (up to 90%) against pesticide exposure (35, 47, 50).

The dermal exposure assessment results presented in this study highlight significant exposure in hand-wash, patch, and wipe samples collected from the farmworkers, due to unsafe pesticide handling practices adopted by them. However, the exposure levels depend on several other factors that might drastically affect the amount of pesticide residue adherence and absorption in the respective farmers' exposed body regions (2). It is further noteworthy to point out that some of the pesticide residues detected, such as acephate, quinalphos, profenofos, chlorpyrifos, imidacloprid, emamectin benzoate, dimethoate, and phenthoate, belong to the moderately hazardous class (II) of toxicity, except monocrotophos, which belongs to a highly hazardous class (Ib), and dimethoate, which is considered to have potential mutagenic properties (38, 51). Furthermore, in the present study, the residues of acephate and monocrotophos, which are reported to have been banned for their use for agricultural purposes in the EU countries, were detected among the farmworkers since they are still available in the outlets for their use, which is a major issue of concern (13, 52). It is further interesting to note that despite the ban on the pesticide phorate for its registration and use by the Insecticide Board of India, residues were detected among the farmworkers in the present study (53). Furthermore, the safety analysis results in terms of MOS also revealed that the handling of 5 out of the 10 pesticides tested was found unsafe, highlighting the risk associated with handling concentrated pesticide mixtures that were not done as per the recommended dosage or due to failure to adopt GAPs.

In addition, the study results on the probabilistic health risk assessment also revealed that out of the various pesticide residues detected in the dermal washings of the farmworkers, the HQ values for four pesticide residues (monocrotophos, quinalphos, profenofos, and chlorpyrifos) were >1 indicating non-cancer risks, while they were <1 for acephate, phorate, dimethoate, emamectin benzoate, and imidacloprid, demonstrating that the chronic risks caused by these pesticide residues were within the tolerance range of humans. Furthermore, the cumulative risk (HI value) for all the detected pesticide residues was above 1, indicating the presence of non-cancer risks associated with pesticide exposure among the farmworkers. The study results are consistent with other studies conducted elsewhere, demonstrating that fifteen of eighteen HQs and the specific HIs values were above 1, indicating various risks from the pesticide mixture (54). Another study also revealed the risk assessment associated with dermal exposure to chlorpyrifos, indicating a high risk among applicators with HQ values ranging from 1.5 to 9 of causing acute adverse health effects (55).

## 5. Conclusion

As such, the available studies on dermal exposure using patch dosimeter, wipe, and hand-washing methods, assessment of health risk using HQ and HI indices as safety measures, and impact of the use of PPE on minimization of exposure using a sensitive and selective validated multi-residue method using LC-MS/MS among Indian farmers in a real-time field scenario are limited. Study results revealed higher concentrations of pesticide residues in the hand-washing samples, followed by wipe samples, implying that the distribution of contamination was particularly high in the hands' region, followed by the face/neck region. Risk assessment is an essential tool for estimating the likelihood of adverse effects to which individuals are exposed so as to identify the need for initiating preventive measures. Risk assessment in the present study based on the cumulative HQ (HI) was above 1, implicating the presence of non-carcinogenic risks associated with dermal exposure to pesticides among farmworkers. However, most of the pesticide residues detected in the dermal washings are categorized as endocrine-disrupting chemicals and have an impact on other biochemical parameters such as reproductive hormones, immunological parameters, cholinesterase activity, inflammatory markers, nutrients, and liver function, which are reported in our earlier studies (31, 56, 57). Additionally, the unsafe handling practices of pesticides, reflected in terms of low MOS values, were found to be a better/suitable indicator for evaluating the risk. Furthermore, the evaluation of dermal exposure after using the PPE provided to them indicated lower pesticide residue levels, highlighting the need for the use of adequate PPE as an important parameter from the farmers' safety point of view. The present study findings could be used as a surrogate for the assessment of dermal pesticide exposure among farming groups under similar pesticide use scenarios in other geographical areas and climatic conditions. This may also aid in the creation of databases for risk assessment *via* dermal penetration or absorption, highlighting the necessity of thorough education and training programs to raise awareness for a better comprehensive understanding of safe handling procedures to protect the farming community from exposure. Furthermore, there is also a need for more detailed studies using a larger sample size to comprehensively measure occupational exposure to pesticides in order to evaluate the health risks in multiple exposure routes and scenarios and develop appropriate prevention strategies within the affordable range of the farmworkers. These data generated would facilitate the policymakers and Government Agencies not only to strengthen the guidelines by revising and updating them so as to make PPE use mandatory and also to make it accessible at an affordable cost to the farming community and further advise them to adopt the GAPs both from their health point of view and the nation's interest.

## Data availability statement

The raw data supporting the conclusions of this article will be made available by the authors, without undue reservation.

## Ethics statement

The studies involving humans were approved by the Ethics Committee of the Indian Council of Medical Research - National Institute of Nutrition, Hyderabad, India (REF NIN Protocol number 11/I/2016). The studies were conducted in accordance with the local legislation and institutional requirements. The participants provided their written informed consent to participate in this study.

## Author contributions

SL was involved in the analysis of the data, interpretation of the results, and wrote the first draft of the manuscript. PJ contributed to the conception, design of the study, and edited the whole manuscript. BJ reviewed and edited the manuscript, provided critical inputs on data analysis, and data interpretation using appropriate statistical analysis. PY, AP, JV, and MN carried out the fieldwork, collection, and processing of the samples. SK has done the statistical analysis. All authors contributed to the manuscript revision, read, and approved the final version to be submitted.

## Supplementary material

Supplementary File 1 Questionnaire administered to the farm-workers.

Supplementary Table S1 Toxicity parameters for detected pesticides.

Supplementary Table S2 Optimized mass parameters for organophosphorous compounds in multiple reaction monitoring (MRM) mode using different energy profiles.

Supplementary Table S3 Quality control parameters of the LC-MS/MS method for determination of pesticide residues in hand-washing.

Supplementary Table S4 Quality control parameters of the LC-MS/MS method for determination of pesticide residues in patch/wipe.
