# Supplementary material for: Mitigation of pesticide residue levels in the exposed dermal regions of occupationally exposed farmworkers by use of personal protective equipment
Source: Front Public Health. 2023 Aug 31;11:1232149. doi: 10.3389/fpubh.2023.1232149 (PMC10502222; doi:10.3389/fpubh.2023.1232149)
Supplement: Supplementary File 1 — Questionnaire administered to the farm-workers. [file Data_Sheet_1.PDF]

DERMAL PENETRATION OF PESTICIDE RESIDUES IN FARM WOMEN WORKERS: ASSESSMENT OF PROTECTIVE GEAR AS A PREVENTIVE MEASURE

SCHEDULE NO.:

DATE OF SURVEY: [| | | | |]

DD MM YY

1. **NAME OF THE INDIVIDUAL:** ....

   Name of Father/Husband : ………………………………………………

2. **Age** (years) [ ] **3. Gender:** 1. Male 2. Female [ ]

DEMOGRAPHIC PARTICULARS:

4. **Village & Mandal** ....

5. **District.** ....

6. **Ind./No** [ ]

METROLOGICAL DATA:

7. **Relative humidity** (µl/min) [ ]
8. **Temperature** (°C) [ ]
9. **Wind velocity** (m/s) [ ]

10. **Wind direction** ……………….

11. Weight (Kg) [ ] **12. Height** (cm) [ ] **13. Body area** (dm^2) [ ] **14. Body temperature** (°C) [ ]

15. MEASUREMENTS OF PULSE RATE/BLOOD PRESSURE:

Pulse/minute [| |]

**Blood pressure:**	**Systolic**		**Diastolic**
I	[| |]	/	[| |]
II	[| |]	/	[| |]
III	[| |]	/	[| |]

16. BLOOD GLUCOSE LEVELS: ………….mg/dL

17. PRIMARY HAND: 1. Right 2. Left 3. Ambidextrous (both hands) ☐

18. LOCATION OF HOUSE

1. In the farm
2. Away from the farm

☐

19. EDUCATIONAL STATUS

1) Illiterate	4) Secondary (6th -10th)	☐
2) Write	5) Inter	
3) Primary (1-5th)	6) Degree & above	

20. MAJOR OCCUPATION

1. Agriculture
2. Agriculture labour
3. Other labour
4. Others (specify)

☐

21. EXTENT OF LAND HOLDING (IN ACRES) ……………..

PARTICULARS OF EXPOSURE TO PESTICIDE SPRAYING ACTIVITY

22. TYPE OF WORK

1. Mixing 2. Loading 3. Application/spraying 4. All specified ☐

23. DURATION OF MIXING, LOADING AND APPLICATION (MLA) ACTIVITIES

During the past year (months or years): ☐

24. HOURS SPENT FOR MLA THE FORMULATION (AVERAGE): ☐ Hours

25. APPLICATION MODE?

1. Hand sprayer 2. Tractor sprayer ☐

26. SPRAYER TYPE?

1. Knapsack/Backpack with hand pump
2. Automisator (electrical)
3. Backpack pump (petrol regulated)
4. Nebulisator (hand bottle spray)
5. Any other? Specify…..

☐

27. SIZE OF THE SPRAY TANK …………. (Liters or Hectoliters)

28. HOW OLD IS THE SPRAY EQUIPMENT? ……… (months/years)

29. WHAT IS THE TYPE OF CONNECTING PIPE TO SPRAY TANK?

1. Polypropylene pipe
2. Polyurethane pipe
3. Nylon pipe
4. HDPE pipe
5. PVC pipe
6. Steel pipe
7. Stainless steel pipe
8. Rust proof Steel pipe
9. Any other, specify…………………..

☐

30. WHAT IS THE LIFE SPAN OF SPRAY TANK IN GENERAL? …………. (Months/years)

31. ARE THE SPARE PARTS CHANGEABLE/REPLACEABLE? 1) Yes 2) No

32. HOW OFTEN THE SPRAY TANKS OR SPARE PARTS ARE REPLACED? …………….

33. FUNCTIONING OF THE SPRAY TANKS

1. Any damage or leakage to spray tank
2. Leakage through nozzles
3. Any damage to the connecting pipes
4. All specified

34. ARE THESE DAMAGE REPAIRED?

1) Yes 2) No

35. DOES ANY ACCIDENT/INCIDENT (SPILLS OR SPLASHES) HAPPEN DURING MLA PHASE?

1) Yes 2) No

36. OTHER THAN MLA THE FORMULATION, OTHER JOBS CARRIED OUT DURING LAST ONE YEAR

1. Preparation of the soil 2. Sowing 3. Watering 4. Thrashing
5. Cutting 6. Harvesting 7. Removing weeds 8. Cleaning & maintenance
9. Re-entry into the sprayed fields (After how many days? ………………..) 10. All of above

Any other activity in the farm, specify ……………………………

37. IF RE-ENTERING INTO THE SPRAYED FIELDS, ARE YOU USING ANY PROTECTIVE DEVICE? 1. Yes 2. No 3. NA

If yes, which protective device? ……………………

38. THE MAJOR CROPS UNDER CULTIVATION

Crop	1. Paddy	2. Cotton	3. Chilli	4. Tomato	5. Brinjal	6. Okra
1. Yes 2. No						

Any other, specify……………………………………………………………………………….

SCHEDULE FOR MLA THE FORMULATIONS OF PESTICIDES FOR SPRAYING ON TO THE SELECTED CROPS

Crop	1. Paddy	2. Cotton	3. Chilli	4. Tomato	5. Brinjal	6. Okra
**39. Duration (months)**						
**40. HOW MANY ROUNDS OF PESTICIDES SPRAY PER SEASON?** (rounds)						

41. PARTICULARS OF EXPOSURE WHILE MLA THE FORMULATIONS OF SPECIFIC PESTICIDES IN THE PAST ONE YEAR IN THE FARM
(by the respondent) (Enter 9 NA)

Name of the pesticide/product/mixture	Active substance and its concentration (%)	Amount of product added per tank (kg)	If yes, particulars of pesticides used		Solvents used for mixing formulations	Frequency of use of mixing the formulation		
			(Formulation)<br>1. Solid, 3. Not sure<br>2. Liquid,	Used as 1. Insecticide<br>2. Herbicide<br>3. Fungicide<br>4. Rodenticide<br>5. Not sure		No. of Hours/day	No. of days/month	No. of months/year

42. DID YOU EXPERIENCE ANY OF THE FOLLOWING MORBIDITY/MORATLITY SYMPTOMS WHILE DURING/AFTER MLA THE FORMULATION OF PESTICIDES AND/OR ANY SPECIFIC PESTICIDE:

Type of pesticide exposed to: ☐ ☐ ☐ **Duration of exposure: days** ☐☐ ☐☐ ☐☐

	**Morbidity**<br>1.Yes<br>2. No<br>9. NA	**If yes, how many hours after exposure**<br>1. Immediately<br>2. <24 hrs<br>3. 24-48 hrs.<br>4. 48-72 hrs	**Duration of sickness**<br>1. < week 6. 6-9 months<br>2. 1-3 weeks 7. 9-12 months<br>3. 3-4 weeks 8. Still persisting<br>4. 1-3 months 9. NA<br>5. 3-6 months		**Morbidity**<br>1.Yes<br>2. No<br>9. NA	**If yes, how many hours after exposure**<br>1. Immediately<br>2. <24 hrs<br>3. 24-48 hrs.<br>4. 48-72 hrs	**Duration of sickness**<br>1. < week 6. 6-9 months<br>2. 1-3 weeks 7. 9-12 months<br>3. 3-4 weeks 8. Still persisting<br>4. 1-3 months 9. NA<br>5. 3-6 months
Salivation				Difficult in breathing			
Nausea				Red eyes			
Vomiting				Burning sensation in eyes			
Cough				Itching sensation			
Abd. Pain				Lacrimation			
Diarrhea				Blurred Vision			
Headache				Fatigue			
Confusion				Weakness			
Dizziness				Tingling of extremities/fingers/ toes			
Fainting				Muscle Twitching			
Coma				Convulsion			
Running nose				Paralysis			
Chest tightness				Itching of skin			

43. DO YOU SUFFER FROM ANY DEGENERATIVE/CHRONIC DISORDERS? 1. Yes 2. No

1. Cardiovascular 2. Hypertension 3. Hypotension 4. Diabetics 5. Tuberculosis 6. Asthma 7. Cancer

44. DO YOU HAVE ANY DERMATOLOGICAL PROBLEM? 1) Yes 2) No

1. Allergic rash 2. Prickly heat 3. Peeling of skin 4. Any other, specify………….. 9. NA

45. WERE YOU TREATED FOR THE ABOVE? 1. Yes 2. No 9. NA

46. IF YES, WHERE WERE YOU TREATED?
1. At home 2. PHC
3. Private Clinic 4. Govt. Hospital

47. IF HOSPITALIZED, FOR HOW MANY DAYS?
Enter 00 if not hospitalized, 98 if more than 99 days

48. SYSTEM OF MEDICINE
1. Ayurveda 3. Siddha
2. Homeopathy 4. Allopathy 5. Home remedies (specify)
9. NA

PERSONAL HABITS

49. Dietary habits

1. Pure vegetarian
2. Lacto-vegetarian
3. Lacto-ovo-vegetarian
4. Non-vegetarian

USE OF ALCOHOLIC BEVERAGES/SMOKING

50. CONSUMPTION OF ALCOHOLIC BEVERAGES 1. Yes 2. No 3. Ex

51. FREQUENCY OF DRINKING?

1. Daily
2. Twice/thrice a week
3. Once a week
4. Occasionally

52. WHAT TYPE OF BEVERAGE?

1. Local (toddy, araq or any other)
2. Beer
3. Whiskey
4. Any other, specify ……………….

53. AMOUNT OF BEVERAGE PER DRINK …………mL

54. SMOKING STATUS 1. Yes 2. No 3. Ex

55. FREQUENCY OF SMOKING?

1. Daily
2. Twice/thrice a week
3. Once a week
4. Occasionally

56. NUMBER OF CIGARETTES PER DAY? …………

KNOWLEDGE AND PRACTICE WHILE MLA THE FORMULATIONS OF PESTICIDE USE:

57. KNOWLEDGE ABOUT THE ROUTE OF EXPOSURE 1. Yes 2. No

1. Inhalation 2. Skin 3. Oral 4. Eye 5. All

DETAILS ON TYPE OF CLOTHING..

DETAILS OF PERSONAL PROTECTIVE EQUIPMENT (PPE) USAGE:

58. HOW DO YOU MIX THE PESTICIDES?

1. Bare handed 2. With gloves on 3. With aid of wooden stick or metal rod

4. Any other mode

59. DO YOU USE ANY PROTECTIVE DEVICES WHILE MLA ACTIVITIES OF PESTICIDE FORMULATIONS?

1. Yes 2. No ☐

IF YES, WHAT DO YOU USE?

60. GLOVES? 1. Yes 2. No 3. Not aware ☐

1. Latex
2. Rubber
3. Neoprene

☐

61. RESPIRATORY PROTECTIVE EQUIPMENT 1. Yes 2. No 3. Not aware ☐

1. N-series/P-series/Dust mask with Assigned Protection Factor (APF)
2. Handkerchief as mask
3. Any other, specify…………

☐

62. APRON 1. Yes 2. No 3. Not aware ☐

1. Chemical resistant apron
2. Full body apron
3. Full sleeves shirt
4. Half sleeves shirt/T-shirt

☐

63. HEADGEAR PROTECTION 1. Yes 2. No 3. Not aware ☐

1. Chemical resistant headgear
2. Normal cap/ headgear
3. Towel/ scarf

☐

64. GOGGLES 1. Yes 2. No 3. Not aware ☐

1. Goggles with shields at the front, brow, and temple
2. Goggles without shields
3. Any other, specify…………

☐

65. PROTECTIVE SHOES 1. Yes 2. No 3. Not aware ☐

1. Chemical resistant protective rubber/gum boots
2. Normal gum boots
3. Any other, specify…………

☐

66. DO YOU THINK THE ABOVE DEVICES PROTECT YOU? 1. Yes 2. No 9. NA ☐

67. IF NO WHY?

1. Inconvenient to wear protective devices 2. Feel suffocated if wear the protective devices 3. Heat stress
4. No access to wear protective devices 5. Cannot judge whether mixing is proper or not with gloves on
6. Any other reason, specify:.. ☐

CONDITION OF PROTECTIVE DEVICES USED:

68. IF ANY DAMAGE HAPPEN TO ANY OF THE PROTECTIVE DEVICES (TORN/ DIRTY/PESTICIDE SPILLS/OPEN SEAMS OR JOINTS IN APRON), HOW FREQUENTLY THEY ARE REPLACED OR WASHED? ....

69. THE PROTECTIVE DEVICES ARE?

1) Maintained 2) Not- maintained

DETAILS OF PRECAUTIONS FOLLOWED WHILE HANDLING PESTICIDES:

70. ARE YOU AWARE THAT THERE ARE SYMBOLS ON THE TIN / BOTTLE/ PACK OF PESTICIDES IN THE LEVEL OF TOXICITY? 1. Yes 2. No 9. Not aware

71. IF YES, WHAT ARE THEY?

1. Red- Extremely toxic 2. Yellow- Highly toxic 3. Blue - Moderately toxic 4. Green – less toxic 9. NA

72. DO YOU KNOW ABOUT HARMFUL EFFECTS OF PESTICIDE EXPOSURE? 1. Yes, 2. No 3. Not aware

73. DO YOU READ THE PRECAUTIONS ON THE LABEL?

1. Always 2. Some times 3. Rarely 4. Never

74. IF NO, WHY?

1. Cannot read 2. Never thought of it 3. Other reasons 9. NA

Specify: ....

75. WHAT IS THE SOURCE OF INFORMATION ABOUT PESTICIDES?

1. Retailers 2. Other farmers 3. Krishi-Vigyan kendra 4. Agricultural official or any other agricultural authority

5. Any other source, specify………………

76. PRECAUTIONS NORMALLY TAKEN WHILE MLA PESTICIDE FORMULATIONS?

1. Yes 2. No 9. NA

77. IF YES?

1. Wash hands immediately after use 2. Take bath immediately after use

3. Change work clothes immediately/daily 4. Wash work clothes immediately/ daily

78. WHERE DO YOU STORE THE PESTICIDES?

1. In the farm 2. In the house in a separate room 3. In the house along with others

4. Any other place, specify...

KNOWLEDGE & PRACTICES WITH REGARD TO EXPOSURE TO PESTICIDE:

79. IF PESTICIDE ACCIDENTALLY FALLS ON YOUR CLOTHS, WHAT DO YOU DO? ☐

1. Change cloths immediately 2. Wash after going home 3. Reuse without washing 4. Others (specify)…….

80. IF PESTICIDE ACCIDENTALLY FALLS ON YOUR BODY, WHAT DO YOU DO? ☐

1.Wash only with water 2. Wash with soap and water 3. Wash with kerosene 4. Wash with mud/cow dung 5. Apply oil

If yes, When do you do? 1. In the field, immediately after spillage/splash 2. After going home ☐

81. WHERE DO YOU THROW THE EMPTY CONTAINERS OF PESTICIDES? ☐

1. In the agricultural fields 2. In the water bodies 3. In the canal/passage of the agricultural fields

4. In the open/barren fields 5. In the dumping ground where the waste material is dumped 6. Sell as scrap

82. DO YOU USE THE EMPTY CONTAINERS OF PESTICIDES FOR ANY PURPOSE? 1. Yes 2.No ☐

If yes, 1. For filling the drinking water 2. Any other purpose, specify………………………………… ☐

83. DID YOU EVER SEE OR NOTICE YOUR CHILDREN PLAYING WITH THEM? 1. Yes 2.No ☐

If yes, 1. Do they put them in their mouths 2. Any other, specify………………………………. ☐

84. WHILE SPRAYING TASK, WHERE YOU WILL EAT/DRINK? ☐

1. In field near to sprayed crops 2. In field away from sprayed crops

3. Outside field (farmhouse or other place away from field)

FAMILY HISTORY:

85. HISTORY OF MORBIDITY / MORTALITY AMONG FAMILY MEMBERS ATTRIBUTABLE TO PESTICIDE EXPOSURE DURING PAST ONE YEAR? 1.Yes 2. No ☐

If yes, give details:

	Name	Age (Yrs.)	Sex 1. Male 2. female	Pesticide exposed to (codes)	If yes, signs & symptoms (codes)	Treatment availed 1. Yes 2. No
Illness	1.	☐☐	☐	☐		
	2.	☐☐	☐	☐		
	3.	☐☐	☐	☐		
Deaths	Name of the diseased	Age (Yrs.)	Sex	Pesticide exposed to	Signs & symptoms	Treatment availed 1. Yes 2. No
	1.	☐☐	☐	☐		
	2.	☐☐	☐	☐		
	3.	☐☐	☐	☐		

Information collected by: Signature:

Name: Date:
