# Supplementary material for: Mitigation of pesticide residue levels in the exposed dermal regions of occupationally exposed farmworkers by use of personal protective equipment
Source: Front Public Health. 2023 Aug 31;11:1232149. doi: 10.3389/fpubh.2023.1232149 (PMC10502222; doi:10.3389/fpubh.2023.1232149)
Supplement: Supplementary Table S1 — Toxicity parameters for detected pesticides. [file Data_Sheet_2.PDF]

Supplementary Material

Mitigation of pesticide's residues levels in the exposed dermal regions of occupationally exposed farm-workers by use of personal-protective-equipment

Summaiya Lari[1, 2], Janardhan Vanka[1], Babban Jee[3], Arun Pandiyan[1], Praveen Yamagani[1], Senthil Balakrishnan Kumar[1], Mohan Naidu[1] and Padmaja Jonnalagadda[1]*

[1]ICMR-National Institute of Nutrition, Tarnaka, Osmania University, PO, Hyderabad, India

[2]Department of Biochemistry, Acharya Nagarjuna University, Guntur, India

[3]Department of Health Research, Ministry of Health and Family Welfare, Government of India, New Delhi, India

***Corresponding author:**
Padmaja Jonnalagadda
drpadmajaj@gmail.com

1 Supplementary Tables

Table S1. Toxicity parameters for detected pesticides

**Pesticide**	**log K_{ow}**	**Molecular weight (MW)** g mol^{-1}	**Dermal permeation constant (PC)** cm h^{-1}	**ARfD** mg kg^{-1} day^{-1}
**Acephate**	-0.85	183.2	1.4276	0.004
**Monocrotophos**	-0.20	223.16	1.5482	0.002
**Quinalphos**	4.44	298.30	461.5759	0.0005
**Profenofos**	4.68	373.63	111.4803	0.00199
**Chlorpyrifos**	4.96	350.6	633.4877	0.01
**Phorate**	3.56	260.4	69.6524	0.003
**Dimethoate**	0.78	229.3	2.2397	0.0002
**Emamectin benzoate**	5.9	1008.2	1.3523	0.00075
**Imidacloprid**	0.57	255.66	1.8324	0.14
**Phenthoate**	3.69	320.4	27.2266	NA

Source: Pubchem, 2023; OECD, 2011

Table S2. Optimized mass parameters for organophosphorous compounds in multiple reaction monitoring (MRM) mode using different energy profiles

Analyte	MRM Transition (Parent/quantifier)	Ionization mode*	DP	EP	CE	CXP	R_T (min)
Acephate	184/143	+	46	10	11	12	8.05
Monocrotophos	224.1/127.1	+	46	6	21	12	11.87
Quinalphos	299.1/147	+	60	5	30	7	13.21
Profenofos	375/305	+	61	10	27	26	14.62
Chlorpyriphos	350/198	+	56	10	19	8	19.02
Phorate	261/75	+	36	10	15	12	22.02
Emamectin Benzoate	872.6/158.2	+	55	10	20	10	22.39
Dimethoate	230/199	+	51	10	13	18	23.03
Phenthoate	321/135	+	44	8	15	12	23.57
Imidacloprid	255.9/208.9	+	54	10	15	12	24.22
TPP (IS)	327.1/77.1	+	96	8	63	4	22.1

*Ionization was carried out by Electrospray Ionization (ESI) in positive ion mode; Declustering Potential (DP); Entrance Potential (EP); Collision Energy (CE); Collision cell Exit Potential (CXP); Retention time (RT)

Table S3. Quality control parameters of the LC-MS/MS method for determination of pesticide residues in hand-washing

Analyte	LOD	LOQ	R^2	Precision (%RSD)						% Recovery (n=6)	
				Intra-day Rp			Inter-day Rc				
				1 / 5	50	50 0	1 / 5	50	500	50	500
Acephate	0.5	5	0.998	2.1	3.8	3.2	4.3	10.7	3.8	96 ± 1	100 ± 2
Monocrotophos	1	2	0.998	3.3	3.5	5	14.7	6	2	94 ± 2	99 ± 3
Quinalphos	0.5	1	0.996	6.1	2.7	4.3	11.7	8.5	8.4	96 ± 1	97 ± 2
Profenofos	0.5	1	0.998	7.3	6.7	2.8	8.4	3.7	6.2	96 ± 3	98 ± 1
Chlorpyriphos	1	2	0.999	4.6	3.7	7.4	10.2	7.2	6.8	95 ± 3	79 ± 4
Phorate	0.5	1	0.998	3	7.2	3	8.3	15.5	13	95 ± 3	99 ± 4
Dimethoate	1	2	0.999	3.2	3.9	4.7	15.3	7	8.2	85 ± 1	94 ± 3
Emamectin Benzoate	1	5	0.987	3.8	1.6	3.6	7.6	4.8	15	99 ± 1	99 ± 2
Imidacloprid	1	5	0.999	5.1	2.3	6.3	15.4	14.2	11. 3	97 ± 2	85 ± 4
Phenthoate	1	2	0.999	3.7	4.4	4.1	10.5	12	4	101 ± 3	94 ± 3

Values in ng mL-1; Correlation coefficient (R); Rp: Repeatability (n=6); Rc: Reproducibility (n=6); SD: Standard deviation; RSD: Relative standard deviation

Table S4. Quality control parameters of the LC-MS/MS method for determination of pesticide residues in patch/wipe

Analyte	LOD	LOQ	R^2	Precision (%RSD)						% Recovery (n=6)	
				Intra-day Rp			Inter-day Rc				
				1 / 5	50	500	1 / 5	50	500	50	500
Acephate	0.2	0.5	0.995	2.7	3.0	1.8	13.3	3.9	4.7	104 ± 3	104 ± 2
Monocrotophos	0.5	5	0.998	7.6	3.3	2.9	7.2	12.6	10.9	104 ± 2	100 ± 3
Quinalphos	0.2	0.5	0.997	1.6	3.9	4.3	5.6	11.1	9.9	100 ± 3	101 ± 3
Profenofos	0.5	5	0.991	4.6	2.3	3.3	10.2	11.5	9.8	102 ± 2	100 ± 2
Chlorpyriphos	0.5	1	0.999	1.6	3.6	1.3	7.7	10.9	14.0	99 ± 3	98 ± 3
Phorate	0.5	5	0.999	3.9	5.8	3.6	17.4	18.2	13.3	99 ± 4	86 ± 2
Dimethoate	0.2	0.5	0.999	0.8	2.2	2.9	14.1	10.2	13.1	103 ± 1	90 ± 3
Emamectin Benzoate	0.5	5	0.995	5.1	4.7	4.3	10.5	14.7	5.8	95 ± 4	100 ± 3
Imidacloprid	0.5	5	0.999	0.6	4.0	2.2	11.2	14.8	14.5	100 ± 4	96 ± 2
Phenthoate	0.5	1	0.999	3.9	3.3	2.8	10.8	4.9	13.0	97 ± 3	98 ± 2

Values in ng mL-1; Correlation coefficient (R); Rp: Repeatability (n=6); Rc: Reproducibility (n=6); SD: Standard deviation; RSD: Relative standard deviation
